# Unresponsiveness to Glucantime Treatment in Iranian Cutaneous Leishmaniasis due to Drug-Resistant Leishmania tropica Parasites

**DOI:** 10.1371/journal.pmed.0030162

**Published:** 2006-04-18

**Authors:** Ramtin Hadighi, Mehdi Mohebali, Patrick Boucher, Homa Hajjaran, Ali Khamesipour, Marc Ouellette

**Affiliations:** 1School of Public Health and Institute of Public Health Research, Tehran University of Medical Sciences, Tehran, Iran; **2**Centre de Recherche en Infectiologie and Département de Biologie Médicale, Université Laval, Québec, Canada; **3**Center for Research and Training in Skin Diseases and Leprosy, Tehran University of Medical Sciences, Tehran, Iran; Royal Melbourne HospitalAustralia

## Abstract

**Background:**

Recent circumstantial evidence suggests that an increasing number of Iranian patients with cutaneous leishmaniasis are unresponsive to meglumine antimoniate (Glucantime), the first line of treatment in Iran. This study was designed to determine whether the clinical responses (healing, or non-healing) were correlated with the susceptibility of *Leishmania* parasites to Glucantime.

**Methods and Findings:**

In vitro susceptibility testing was first performed on 185 isolated parasites in the intracellular mouse peritoneal macrophage model. A strong correlation between the clinical outcome and the in vitro effective concentration 50% (EC _50_) values was observed. Parasites derived from patients with non-healing lesions had EC _50_ values at least 4-fold higher than parasites derived from lesions of healing patients. A selection of these strains was typed at the molecular level by pulsed-field gels and by sequencing the pteridine reductase 1 *(PTR1)* gene. These techniques indicated that 28 out of 31 selected strains were Leishmania tropica and that three were *Leishmania major.* The L. major isolates were part of a distinct pulsed-field group, and the L. tropica isolates could be classified in three related additional pulsed-field groups. For each pulsed-field karyotype, we selected sensitive and resistant parasites in which we transfected the firefly luciferase marker to assess further the in vitro susceptibility of field isolates in the monocyte cell line THP1. These determinations confirmed unequivocally that patients with non-healing lesions were infected with L. tropica parasites resistant to Glucantime. Additional characterization of the resistant isolates showed that resistance is stable and can be reversed by buthionine sulfoximine, an inhibitor of glutathione biosynthesis.

**Conclusions:**

To the authors' knowledge, this is the first report of proven resistant parasites contributing to treatment failure for cutaneous leishmaniasis and shows that primary Glucantime-resistant L. tropica field isolates are now frequent in Iran.

## Abstract 2

Parasites isolated from patients with non-healing lesions are frequently resistant to to meglumine antimoniate, the first line of treatment in Iran.

## Introduction

The protozoan parasite *Leishmania* is responsible for several pathologies collectively known as leishmaniases. The cutaneous, mucocutaneous, and visceral leishmaniases are caused by different *Leishmania* species and affect several million individuals worldwide [ 1– 3]. The treatment of leishmaniasis in endemic areas relies on chemotherapy, and in several parts of the world the mainstay remains the pentavalent antimony (SbV)–containing drugs Pentostam and Glucantime. Second-line drugs such as pentamidine or liposomal amphotericin B are less useful owing to problems associated with either toxicity or cost [ 2]. The proven clinical efficacy of the oral drug miltefosine was a major breakthrough for anti *-Leishmania* chemotherapy and has become the first-line drug in north-east India where unresponsiveness to SbV drugs in visceral Leishmania donovani is epidemic [ 4, 5]. Unresponsiveness to SbV may be due to several factors, but in one study it was shown that unresponsiveness in L. donovani isolates was correlated with decreased susceptibility to SbV when tested in an intracellular in vitro assay [ 6]. The emergence of SbV-resistant parasites was expected in north-east India because of the high endemicity of the parasite, the high proportion of treated individuals, and the nature of the anthroponotic (man to man) transmission cycle of L. donovani [ 1].

Both cutaneous and visceral forms of leishmaniasis are endemic in different parts of Iran [ 7, 8]. Anthroponotic cutaneous leishmaniasis (ACL) caused by Leishmania tropica and zoonotic cutaneous leishmaniasis (ZCL) are endemic in many parts of Iran with a high incidence rate [ 9– 12]. Glucantime is the first-line drug for the treatment of all forms of leishmaniasis in Iran. Patients with cutaneous leishmaniasis (CL) lesions not responding to SbV treatment have been reported [ 13], and these patients may require alternative treatments **.** In 2002, an outbreak of ACL occurred in Mashhad, north-east Iran, where almost 4,900 CL cases have been detected by clinical and parasitological methods (Khorassan Health Centers Reports 2000–2002). The results of a cross-sectional study in this area showed that 94.2% of isolates were L. tropica [ 14] and that almost 12% of the patients did not respond to treatment with meglumine antimonate (M. Mohebali, unpublished data). The objective of this study was to determine whether clinical unresponsiveness to SbV in patients with ACL was correlated to parasites with decreased susceptibility to SbV.

## Methods

### Study Design

**Figure 1:**
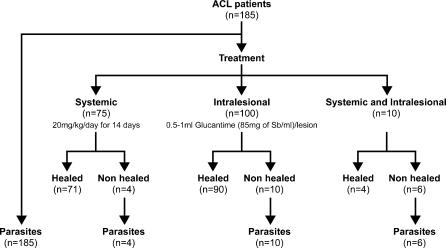
Treatment Outcome and Parasite Isolation Parasites were isolated from 185 patients with suspected ACL prior to treatment. Patients were included in three forks of treatment, and 20 patients did not respond to therapy. Parasites from these nonresponders were also isolated.

Patients of between 20 and 45 y of age and residing in Mashhad (an endemic area of ACL) who were willing to participate were included in the study. The study was approved by the Institutional Ethical Committee of the School of Public Health and Institute of Public Health Research, Tehran University of Medical Sciences. CL was confirmed by parasitological procedures, including observation of the amastigotes under light microscopy with high magnification (×1,000) and their cultivation in specific culture media. Pregnant and lactating patients were excluded from this study. Patients with previous CL history were also excluded. From the 248 patients that were initially included in the study, 185 isolates of *Leishmania* species were collected from skin lesions ( Figure 1). Lesions were in general non-ulcerative (71%), and 63% of patients had single lesions. Patients were treated according to the physician's and/or the patient's decision either systemically, intralesionally, or, for a small number of patients, with both regimens ( Figure 1). The national protocol for the treatment of ACL is systemic SbV (20 mg/kg/d for 14 d), and noncompliance to this protocol is mostly induced by the patient's wish. No interference with common practice implemented in the treatment strategy was made through this study. Successful treatment was defined as complete re-epithelialization of all lesions with no relapse within 6 mo of follow up. Parasites were also collected from skin lesions which did not respond to treatment ( *n* = 20, see Figure 1). Patients were classified as nonresponders when they presented lesions at a 24-mo follow up. These patients were treated with a second course of Glucantime.

### Parasites and Culture

Parasites were isolated from skin lesions as described [ 15] and grown in NNN medium and subcultured in RPMI 1640 medium supplemented with 15% fetal calf serum (FCS). Identification of *Leishmania* parasites was attempted by PCR typing using a random amplified polymorphic DNA (RAPD) technique [ 8, 14] following a published protocol [ 16]. Selected primers for these studies were A4, 5′ AATCGGGCTG; AB1–07, 5′ GGTGACGCAG; 327, 5′ ATACGGCGTC; and 329, 5′ GCGAACCTCC. Species identification of *Leishmania* isolates was further studied by sequencing the *PTR1* gene, a metabolic gene involved in pterin and folate metabolism in *Leishmania* [ 17, 18]. The *PTR1* coding region of each field isolate was amplified by PCR using the primers 5′ CTGCTCCGACCGTGCC and 5′ CCCGGGTAAGGCTGTAGC. The expected 800-bp amplified product was purified and sequenced. The sequences were compared with sequences of *L. tropica, L. major, L. tarentolae,* and L. donovani reference isolates using the ClustalW program ( http://www.ebi.ac.uk/clustalw) and the Molecular Evolutionary Genetics Analysis (MEGA3) software ( http://www.megasoftware.net).

### Pulsed-Field-Gel Karyotyping

Agarose blocks containing *Leishmania* cells were prepared as described [ 19]. Briefly, cells were resuspended in HEPES buffer at a density of 1 × 10 ^8^ cells/ml and mixed with low-melting-point agarose. Cells were lysed in the presence of 0.5 M EDTA (pH 9.5), 1% SLS, and proteinase K, and the chromosomes of *Leishmania* cells were resolved by a BioRad (Hercules, California, United States) contour-clamped homogeneous electric field (CHEF) mapper for separating 0.2–2.0-Mbp DNAs over a period of 28 h. Chromosomes were revealed by ethidium bromide staining.

### Susceptibility Testing

#### Mouse peritoneal macrophages

The macrophages of peritoneal fluid of BALB/c mice were collected and resuspended at 5 × 10 ^4^/ml in RPMI 1640 supplemented with 15% FCS, as described by others [ 6]. Cells were plated in eight-chamber LabTek tissue-culture slides, and adherent macrophages were infected with late-logarithmic promastigote parasites at a parasites-to-macrophage ratio of 4:1. After 2 h of incubation at 34 °C, free promastigotes were removed and Glucantime was added. Each 5-ml ampoule of Glucantime contained 1.5 g meglumine antimoniate corresponding to 0.405 g of pentavalent antimony. The tissue-culture slides were incubated for 3 d, fresh Glucantime was added, and the slides were incubated for an additional 72 h. The slides were fixed and stained with Giemsa [ 20]. Three slides were used for each isolate. The percentage of infected macrophages and the number of parasites per infected cell were evaluated by microscopic examination of at least 100 macrophages. The EC _50_ is defined in this study as the concentration of meglumine antimoniate that reduces the survival of *Leishmania* parasites by 50%. These studies were approved by the Animal Committees of the School of Public Health and Institute of Public Health Research, and of the Center for Research and Training in Skin Diseases and Leprosy, Tehran University of Medical Sciences.

#### THP1 cell line

A number of sensitive and resistant parasites, as determined by the mouse peritoneal macrophage assay, were selected and used for the in vitro susceptibility assay in the human monocyte cell line THP1 [ 21]. Recombinant parasites were produced by transfection of the *Leishmania* isolates with the firefly luciferase–containing vector pSP1.2 LUC αHYGα [ 22] and were used to infect THP1 cells (ratio 20:1) stimulated with phorbol myristic acetate (PMA) as described [ 21]. Luciferase activity was measured after 5 d of incubation in the presence of Glucantime. Previous in vitro and in vivo work demonstrated an excellent correlation between luciferase activity and parasite number [ 20, 21, 23]. Susceptibility to trivalent antimony was also determined in the promastigote stage of the parasites, as described elsewhere [ 24] by measuring the optical density of cultures at 600 nm.

## Results

### Unresponsiveness to Glucantime Treatment

Out of the 248 patients with parasitological proven CL and with no previous history of Glucantime treatment, we grew 185 parasite isolates. The patients were treated either systemically or intralesionally. The two treatment regimens appeared to be equally effective, with no relapse within the first 6 mo after treatment for the majority of patients ( Figure 1). Two responding patients showed relapse after 12 mo, although no work was carried out to determine whether this represented a true relapse or a new infection. Ten patients were first treated intralesionally, but owing to the pain induced by intralesional injection, the patients preferred to receive systemic SbV and therefore received a round of systemic SbV ( Figure 1). Overall, 10.8% of patients (20 out of 185) did not respond to the first course of SbV treatment ( Figure 1). A slightly higher, but not significant, proportion of nonresponding patients were seen in the groups receiving intralesional treatment ( Figure 1).

The goal of this study was to determine whether unresponsiveness to Glucantime is due to the presence of resistant parasites. The EC _50_ of the 185 isolates derived from lesions before initiation of therapy was first measured using the peritoneal mouse-derived macrophage model [ 6]. Similar infection rates were observed between parasites derived from responsive (91.3 ± 12.9 amastigotes/100 untreated macrophages) and unresponsive (83.7 ± 8.0 amastigotes/100 untreated macrophages) patients. The results showed a strong correlation between the clinical outcome and susceptibility values. Indeed, the 165 patients who responded to SbV treatment were infected with parasites with EC _50_ values of less than 10 μg/ml ( *p* < 0.001, as determined by the Mann-Whitney test) ( Table 1). In contrast, the 20 primary nonresponders were infected with parasites that had considerably higher EC _50_ to SbV ratios. These parasites could be identified as falling into two groups: those parasites with intermediate EC _50_ values ranging from 10 to 25 μg/ml and those with EC _50_ values greater than 40 μg SbV/ml ( Tables 1 and 2). These EC _50_ values were obtained from strains isolated prior to the initiation of treatment, but the EC _50_ values of parasites from unresponsive patients were similar regardless of whether the parasites were isolated pre- or posttreatment (unpublished data). Parasites derived from unresponsive patients were, on average, at least four times less susceptible than parasites from responsive patients ( *p* < 0.01, as determined by the Mann-Whitney test ( Table 1).

**Table 1:**
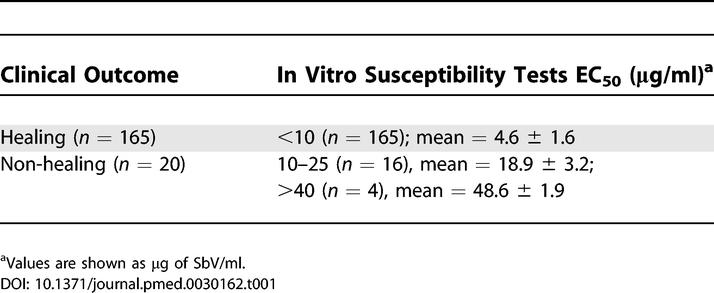
Relationship between Clinical Outcome and In Vitro Susceptibility Testing in Mouse-Derived Macrophages of *Leishmania* Cells Isolated from Patients with ACL

**Table 2:**
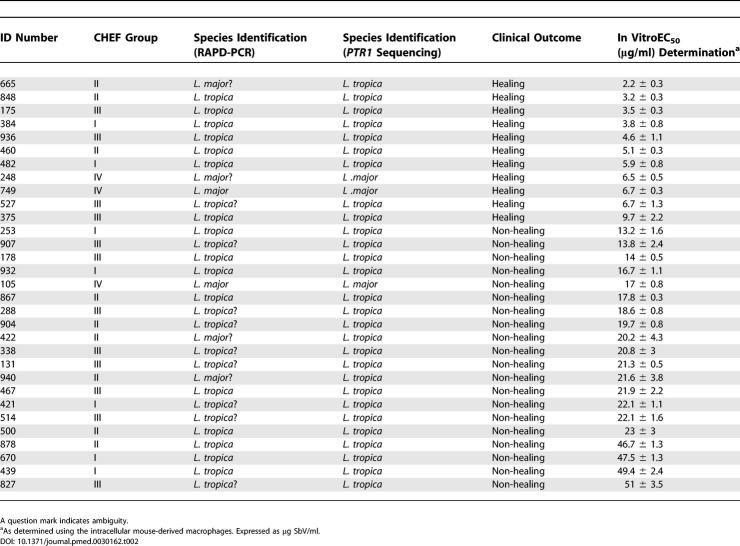
EC _50_ Determination and Characteristics of Selected *Leishmania* Isolates

### Characterization of Parasites

**Figure 2:**
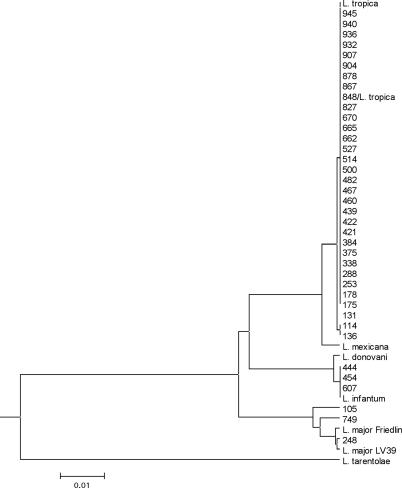
Phylogenetic Analysis of *Leishmania* Isolates Responsible for ACL by Comparing the Sequences of *PTR1* The *PTR1* gene was sequenced in 31 Iranian isolates and control L. major cells (LV39; Friedlin), L. donovani 1S2D, L. infantum (MHOM/67/ITMAP-263), L. tropica ATCC50129, and L. tarentolae TarII. Strain 848 is identical, as determined by RAPD-PCR, to L. tropica MHOM/IR/99, and strain 749 is identical to L. major MHOM/IR/75/ER. Strains 444 and 454 were *Leishmania* parasites isolated from dogs in Iran. The sequences were compared and clustered using the Molecular Evolutionary Genetics Analysis (MEGA3) software.

Further characterization of the parasites was warranted since the observed differences in susceptibility values could be due to the emergence of resistant parasites or to varying clones or species with different intrinsic susceptibilities (see, for example, [ 25]). Thirty-one strains (the 20 nonresponsive and 11 responsive isolates) were characterized at the molecular level by a number of techniques. Attempts to identify the species of parasite were initially carried out using PCR typing by a RAPD technique [ 14]. Unambiguous identification was reached for 18 of the 31 strains, but for the remaining isolates, identification of the species was only tentative ( Table 2). Sequencing of locus is a powerful strategy for microbial species identification, and sequencing of the *PTR1* gene was found to be useful for molecular identification of *Leishmania* species (M. Ouellette, unpublished data). Comparative analysis of the *PTR1* sequences of the 31 isolates showed that most of the selected strains could be identified as *L. tropica,* with three isolates being identified as L. major ( Figure 2). This locus-sequence technique could also resolve any ambiguous results of the PCR-RAPD assay ( Table 2).

**Figure 3:**
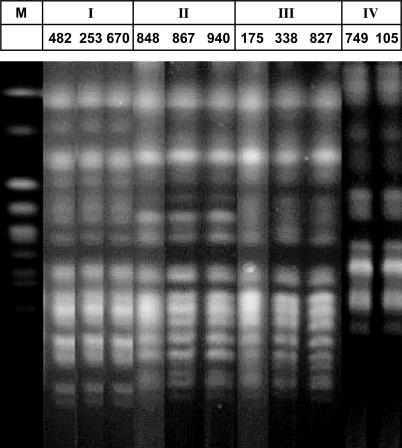
Karyotypes of Iranian *Leishmania* Isolates as Determined by PFGE *Leishmania* cells lysed in situ in agarose blocks were run in agarose gels, and chromosome-sized bands were resolved by CHEF electrophoresis. I, II, III, and IV represent the various karyotypes, and cardinal numbers represent the identification number of *Leishmania* isolates (see Table 2). M indicates S. cerevisiae molecular-weight maker (BioRad).

A pulsed-field-gel electrophoresis (PFGE) technique was used as a second strategy to characterize the 31 selected isolates, and the goal was to find susceptible and resistant parasites with similar karyotypes. Using this strategy, the 31 studied strains were categorized into four PFGE-distinct groups, the most different being group IV which comprised the L. major strains ( Figure 3). Groups I to III consisted of *L. tropica,* and differences in karyotype were small with one to two bands differing ( Figure 3). For L. tropica isolates belonging to Groups I, II, and III, we had a representative isolate that was susceptible, one that was resistant to intermediate levels of SbV, and one that was highly resistant ( Figure 3; Table 3).

**Table 3:**
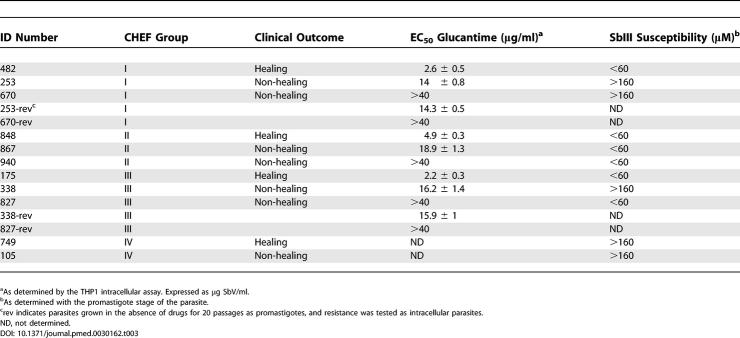
Susceptibility of Parasites to Glucantime and SbIII

**Figure 4:**
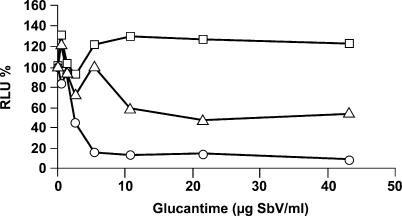
Parasite Susceptibility to Glucantime in the THP1 Cell Line Parasites expressing the firefly luciferase were used to infect THP1, and the levels of parasites surviving Glucantime treatment were calculated by measuring luciferase activity expressed as percentage relative light unit (RLU %). The average of two independent experiments performed in duplicate is shown. Open circles indicate strain 175 (sensitive); open triangles indicate strain 467 (intermediate resistance); and open squares indicate strain 827 (high resistance).

Several susceptible and non-susceptible isolates are genetically closely related ( Figure 3), and further measurements of their susceptibility value using the more tractable system of the THP1 cell line and of luciferase-expressing parasites was warranted. The latter system is a convenient and rapid quantitative method to look at intracellular growth of parasites [ 20]. The firefly luciferase gene as part of the *Leishmania* expression vector pSPαHYGα [ 22] was introduced in L. tropica strains by electroporation. Transfectants resistant to hygromycin were selected and were shown to express the firefly luciferase. These transfectants were used to infect the THP1 cell line, and EC _50_ values in the presence of Glucantime were determined by measuring luciferase activity ( Figure 4). The values obtained with this rapid assay were in excellent agreement with the values obtained using the mouse macrophages assay and Giemsa staining ( Tables 2 and 3). It was not possible to use the luciferase marker with *L. major,* as the available L. major Iranian isolates are intrinsically resistant to hygromycin with an EC _50_ of >5 mM—hence precluding the selection of transfectants using hygromycin.

#### Properties of the resistance phenotype

It is suspected that long-term in vitro growth of patient-derived resistant parasites may lead to a loss of the resistance phenotype, although antimony resistance is stable in parasites selected for resistance in vitro [ 26, 27] or in Indian field isolates [ 28]. The 20 L. tropica strains with intermediate or high resistance levels to Glucantime were grown for at least 20 passages as promastigotes in the absence of drug selection (except for hygromycin B to keep the luciferase marker). A susceptibility assay was carried out in the THP1 cell line, and no decrease in the EC _50_ was observed in these cells compared to cells with a minimum number (between three and five) of passages ( Table 3; unpublished data). While SbV is the drug used against leishmaniasis, there is a general consensus that the active form of the metal is SbIII (reviewed in [ 29]). This metal reduction could occur both in the macrophage [ 30, 31] and in the parasite [ 32– 34]. Promastigote cells selected for SbIII resistance are cross-resistant to SbV as intracellular parasites [ 27, 31]. *Leishmania* parasites selected for SbV resistance are sometimes [ 35, 36], but not always [ 32], cross-resistant to SbIII. We therefore tested the level of susceptibility of the promastigote stage of ACL clinical isolates to SbIII. In some groups of strains (e.g., PFGE group I), the intracellular EC _50_ values toward SbV, and SbIII cross-resistance values as determined in promastigotes, did correlate ( Table 3). For other strains of different PFGE groups, however, there was a lack of correlation between intracellular SbV and promastigote SbIII susceptibilities. The L. major isolates studied were completely insensitive to SbIII as promastigotes ( Table 3).

**Figure 5:**
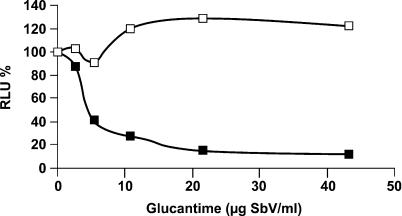
Reversal of Antimony Resistance in Intracellular ACL Strains Using BSO The glutathione-specific inhibitor BSO (5 mM) abrogated resistance in the Glucantime-resistant strains 439, 670, 827, and 878. Only the result for strain 827 (expressing the firefly luciferase) is shown, which represents an average of two duplicate experiments. Open squares indicate strain 827; filled squares indicate strain 827 and BSO.

Several potential mechanisms of resistance to antimonials have been proposed based on work on cells selected for resistance in vitro (reviewed in [ 29]). Intracellular thiols including the glutathione-spermidine conjugate trypanothione [ 26, 37] are important molecules involved in resistance. It has been shown that inhibiting glutathione biosynthesis, a backbone of trypanothione, can lead to antimony resistance reversal in vitro in promastigotes [ 26] or intracellular amastigotes [ 27], as well as in vivo [ 38]. We have shown that buthionine sulfoximine (BSO), an inhibitor of γ-glutamylcysteine synthetase (which is the rate-limiting step of glutathione biosynthesis), can reverse resistance to Glucantime not only in the highly resistant L. tropica isolate 827 ( Figure 5; Table 2) but also in three other studied resistant strains with ID Nos 439, 670, and 878, respectively (unpublished data).

## Discussion

Antimonials have been the pillar of anti- *Leishmania* chemotherapy for more than 60 y. Despite its toxicity, the drug has remained effective and available in endemic countries although, in order to maintain its effectiveness, the drug concentration and duration of therapy have had to increase over the years [ 39], suggesting that the parasites have been slowly acquiring mutations leading to reduced susceptibility to the drugs. However, other factors, such as reduced ability of the human-host immune system to fight the parasites, pharmacological deficiencies, or under-treatment, could also lead to treatment failure [ 40, 41]. In the 1990s, the failure of SbV treatment in north-east India became epidemic [ 4], and in one study it was shown that part of the unresponsiveness in the L. donovani Indian isolates was due to parasites with decreased susceptibility to SbV when tested as intracellular parasites [ 6]. Indeed, there is a general consensus that to be of some relevance, in vitro susceptibility testing has to be done with intracellular stages of *Leishmania* [ 42– 44]. While unresponsiveness to SbV is high in part of India, it is still an effective treatment in several other parts of the world, and it is still the drug of choice for the treatment of ACL in Iran [ 13].

The ACL cycle caused by L. tropica is well established in Iran [ 7, 11, 45], and this human-to-human mode of transmission could facilitate the emergence of parasites less susceptible to SbV. Indeed, anecdotal evidence indicates that an increasing proportion of patients with ACL are failing SbV therapy. In order to test whether resistant isolates exist, we measured the EC _50_ of 185 isolates derived from patients before initiation of treatment. We found a strong correlation between the clinical outcome and the susceptibility levels of parasites ( Table 1). This correlation was highly significant ( *p* < 0.01) as determined by a series of statistical analyses. L. major isolate No. 105, a species usually associated with ZCL, was also found to be resistant to Glucantime ( Table 2). A correlation between susceptibility testing and clinical outcomes, with few outliers, has also been found for L. donovani isolates [ 6] and for L. infantum [ 44]. For the L. infantum isolates, however, this correlation stands only for the short-term outcome and not for the longer-term outcome, possibly because the majority of patients included in this study were infected with HIV-1 [ 44]. The parasites derived from non-healing patients were characterized and were found to be closely related to parasites derived from susceptible patients ( Figure 3; Table 2). To our knowledge, this is the first detailed demonstration that resistant and sensitive clinical *Leishmania* isolates are related and the first proven case of ACL treatment failure due to resistant parasites, strongly pointing to acquired drug resistance in these isolates.

Susceptibility data suggest at least two resistance mechanisms, one leading to an intermediate level of resistance (as found in the majority of the isolates) and the other leading to a high level of resistance. It remains to be explored whether intermediate resistance is a necessary step before achieving high resistance, or whether the mutations arise independently. If resistance was at one point associated with a fitness cost, it would appear that the parasite did compensate for it, since its infectiousness to macrophages was found to be comparable to its survival as an intracellular parasite. Whatever the nature of the mutation, it appears (at least in some cells) to be stable, since parasites grown for more than 20 passages in the absence of drugs still kept the same level of resistance. Similar conclusions about infectivity of resistant cells and stability of the resistance phenotype were also reached for L. donovani clinical isolates [ 28, 46]. The stability of the resistance phenotype would rule against gene-amplification events, as amplicons present in drug-resistant parasites are usually rapidly lost during growth of the parasites in the absence of selection [ 19]. Consistent with this premise, we failed to detect gene amplification in the resistant isolates (R. Hadighi and M. Ouellette, unpublished data) when using a number of techniques enabling the detection of amplified genes [ 47]. Modulation in RNA levels are also commonly associated with drug resistance [ 27, 48– 51]. These changes in RNA expression are usually stable, and this type of change or a point mutation could possibly explain the resistance phenotype found in resistant isolates.

To correlate treatment outcome with susceptibility of the parasites, we used two models, i.e., the mouse-derived macrophages and Giemsa staining and a human monocyte cell line (THP1) and luciferase-expressing parasites. An excellent correlation was found between the two models ( Tables 2 and 3). The second assay system has obvious advantages as it neither requires animals nor involves the labor-intensive parasite staining and counting [ 20, 21]. The required transfection of the parasites is now a relatively straightforward technique that does not appear to modify the properties of the parasite—at least the ones related to macrophage infection and drug susceptibility ( Tables 2 and 3). Comparable results of susceptibility testing using transfected parasites were also obtained for L. donovani field isolates [ 28, 46, 52]. The susceptibility of promastigotes to SbIII did not correlate well with intracellular SbV susceptibility data ( Table 3). This could be due to either different resistance mechanisms, some giving cross-resistance to SbIII, or to varying levels of intrinsic resistance. In addition, this simpler SbIII-susceptibility assay could not be used to predict clinical outcome.

Our report describes, in detail, several L. tropica and one L. major isolates that appear to have acquired mutations leading to decreased susceptibility to SbV and to unresponsiveness towards clinical therapy. It is salient to point out that four out of the 20 primary unresponsive patients responded to a second course of Glucantime treatment. All four harbored parasites with intermediate levels of resistance (EC _50_ 10–25 μg/ml). The next challenge will be to determine the exact molecular mechanisms of resistance. It is possible that, as in the in vitro isolates, thiols are important for the resistance phenotype ( Figure 5). Indeed, preliminary results suggest that glutathione levels are higher in some resistant isolates (G. Roy and M. Ouellette, unpublished data). This suggests a strategy of using drug combinations to reverse drug resistance in the field. Ongoing studies should lead to a better understanding of the molecular mechanisms of antimony resistance in field isolates.

The equation between chemotherapeutic resistance (either in infectious diseases or oncology), as determined by in vitro testing, and treatment failure is intuitively expected but in some occasions this is still unclear. For example, in the case of pneumonia caused by *Streptococcus pneumoniae,* often in vitro findings do not appear to be predictive of in vivo outcome, except with the most highly resistant bacteria (reviewed in [ 53]). However, several reports indicate that an increase in drug resistance is associated with an increased treatment failure (reviewed in [ 54, 55]). Failure in SbV treatment in *Leishmania* has been attributed to several factors other than resistant parasites, including host immunological status, suboptimal treatment, and pharmacokinetic properties [ 40, 41], but it is now clear that an increase in resistance can lead to treatment failure for both visceral leishmaniasis [ 6] and CL. Future work should lead to a precise understanding of the resistance mechanisms in field isolates, which should lead to strategies for rapidly diagnosing resistance in order to improve the clinical management of leishmaniasis.

## Supporting Information

Alternative Language Abstract S1 Translation of the Abstract into French by Marc Ouellette (23 KB DOC)

Alternative Language Abstract S2 Translation of the Abstract into Farsi by Ramtin Hadighi (53 KB DOC)

**Patient Summary**

**Background**

Leishmaniases are a group of diseases caused by different species of the *Leishmania* group of parasites. The diseases are common in many tropical and subtropical areas, where patients are infected through the bite of sandflies that had previously bitten an infected animal or human. The most common form of the disease is cutaneous leishmaniasis, characterized by skin ulcers that take months to heal and often leave ugly and disabling scars. They can also lead to secondary, more dangerous, infections. In most parts of the world, so-called pentavalent antimonials are the drug of choice. Over the past few years, these drugs are becoming less and less effective against the disease, with increasing numbers of patients not responding, showing only partial recovery, or needing higher doses of the drugs to get better. A few additional drugs are now available and are being developed, but some are expensive and others have serious side effects.

**Why Was This Study Done?**

There are several possible explanations as to why the pentavalent antimonials are becoming less effective: parasites could have become increasingly resistant to the drugs, the patients' immune systems could have become weaker over time, or different strains of parasites against which the drugs are not as effective could be responsible for more of the recent cases. It is important for health officials to know which of these apply when they decide on strategies to improve the situation. Results from north-east India, where L. donovani is the predominant cause of the disease, suggest that the parasites are becoming resistant. The scientists who did this study wanted to find out which of these possibilities might be true in Iran, a country in which cutaneous leishmaniasis is common.

**What Did the Researchers Do and Find?**

They discussed the study with many patients with cutaneous leishmaniasis in Mashad, an area in Iran where the disease is common. Of those patients, 248 were suitable and agreed to participate. The researchers then isolated parasites from 185 different ulcerous wounds, including 20 from patients who did not respond well to treatment with pentavalent antimonials. They studied these parasites in various ways. They found that all of the parasites from the 20 nonresponsive patients were either partially or fully resistant to the drugs, meaning that either higher doses of drugs were necessary or that even very high doses could not kill the parasites. They then wanted to know which *Leishmania* species the responsive and nonresponsive parasites belonged to. To answer this question, they determined the genetic code for one specific gene which is known to have different versions in different species. This showed that 19 of the unresponsive isolates belonged to L. tropica and one to *L. major.* Two of the responsive isolates belonged to *L. major,* with the other nine belonging to *L. tropica.* Among the L. tropica isolates, the responsive and nonresponsive isolates showed some similarities at a broader genetic level.

**What Do These Findings Mean?**

The results show that L. tropica parasites can become resistant to pentavalent antimonials. They also suggest that such resistant strains are largely responsible for the decreased effectiveness of these drugs against cutaneous leishmaniasis in Iran. This means that Iran and other countries where resistance is becoming more common need to develop treatment strategies for patients who do not respond to pentavalent antimonials. For researchers, the goal is to identify the molecular mechanisms underlying drug resistance so that they can be taken into account when advising on alternative therapies and developing new drugs against the leishmaniases.

**Where Can I Get More Information Online?**

Here are listed several Web sites with information on leishmaniasis.

World Health Organization:

http://www.who.int/leishmaniasis/en

MedlinePlus:

http://www.nlm.nih.gov/medlineplus/leishmaniasis.html

http://www.nlm.nih.gov/medlineplus/druginfo/uspdi/202733.html (for a description on meglutime antimoniate)

Wellcome Trust Sanger Institute:

http://www.sanger.ac.uk/Projects/L_major

## Abbreviations

ACL: anthroponotic cutaneous leishmaniasis
BSO: buthionine sulfoximine
CHEF: contour-clamped homogeneous electric field
CL: cutaneous leishmaniasis
EC _50_: effective concentration 50%
FCS: fetal calf serum
PFGE: pulsed-field-gel electrophoresis
PMA: phorbol myristic acetate
RAPD: random amplified polymorphic DNA
ZCL: zoonotic cutaneous leishmaniasis
