# Supplementary material for: Unresponsiveness to Glucantime Treatment in Iranian Cutaneous Leishmaniasis due to Drug-Resistant Leishmania tropica Parasites
Source: PLoS Med. 2006 Apr 18;3(5):e162. doi: 10.1371/journal.pmed.0030162 (PMC1435779; doi:10.1371/journal.pmed.0030162)
Supplement: Supplementary file 1 [file pmed.0030162.sd001.doc]

Résistance au Glucantime et échecs thérapeutiques dans le cas de leishmanioses cutanées en Iran.

Ramtin Hadighi ^1^, Mehdi Mohebali ^1 *^, Patrick Boucher ^2^, Homa Hajjaran ^1^, Ali Khamesipour ^3^ et Marc Ouellette ^2 *^.

^1^ School of Public Health and Institute of Public Health Research, Tehran University of Medical Sciences, Tehran, Iran, ^2^ Centre de Recherche en Infectiologie and Département de Biologie Médicale, Université Laval, Québec, Canada, ^3^ Center for Research and Training in Skin Diseases and Leprosy, Tehran University of Medical Sciences.

**Objectif.** Plusieurs évidences circonstancielles récentes suggèrent une augmentation du nombre de patients iraniens atteints de leishmanioses cutanées qui ne répondent plus au méglumine d’antimoniate (Glucantime), toujours le médicament de première indication en Iran. Cette étude avait pour but de corréler la réponse clinique (cicatrisation des lésions cutanées ou pas) avec la sensibilité du parasite *Leishmania* au Glucantime.

**Méthodes et Résultats.** Un panel de 185 souches cliniques ont été testées in vitro pour leur sensibilité au Glucantime suite à l’infection de macrophages murins. Une très forte corrélation a été observée entre la réponse clinique et les mesures de sensibilité. En effet, les parasites isolés de patients avec des lésions qui ne se cicatrisaient pas étaient en moyenne 4 fois moins sensible que les parasites provenant de patients qui ont répondu au traitement. Plusieurs des 185 souches ont été caractérisées en champs pulsés et en déterminant la séquence en nucléotides du gène de la ptéridine réductase 1 (PTR1). La majorité des souches (28/31) a été identifiée comme étant *Leishmania tropica* et 3 comme *Leishmania major*. Les souches de *L. major* avaient un caryotype en champs pulsés différents des *L. tropica*. Les souches appartenant à cette dernière espèce pouvaient être séparées en trois profils de champs pulsés apparentés. Un plasmide contenant le gène rapporteur de la luciférase a été introduit par électroporation dans des souches de *L. tropica* sensibles et résistants au Glucantime. Ces parasites recombinants ont permis de mieux définir la sensibilité in vitro des parasites à l’aide de la lignée cellulaire humaine THP-1. Ces mesures ont permis de confirmer sans équivoque que les patients ne répondant pas au Glucantime étaient infectés par des parasites résistants. Des études plus approfondies ont enfin permis de démontrer que la résistance était un phénotype stable mais qu’elle pouvait être annulée en utilisant un inhibiteur de la biosynthèse du glutathion.

**Conclusions.** Cette étude démontre pour la première fois que des parasites résistants peuvent contribuer à un échec thérapeutique dans le cas de leishmanioses cutanées. Cette étude montre également que la résistance au Glucantime chez *L. tropica* est maintenant prévalente en Iran.
