# Supplementary material for: Unresponsiveness to Glucantime Treatment in Iranian Cutaneous Leishmaniasis due to Drug-Resistant Leishmania tropica Parasites
Source: PLoS Med. 2006 Apr 18;3(5):e162. doi: 10.1371/journal.pmed.0030162 (PMC1435779; doi:10.1371/journal.pmed.0030162)
Supplement: Supplementary file 2 [file pmed.0030162.sd002.doc]

**عدم پاسخ به درمان با گلوکانتیم در لیشمانیوز جلدی ناشی از *لیشمانیا تروپیکا*ی مقاوم در ایران.**

**رامتین حدیقی^1^  مهدی محبعلی^1*^  پاتریک بوچر ^2^  هما حجاران ^1^  علی خامسی پور^3^ و مارک اوولت 2*.**

1- **دانشکده بهداشت و انستیتو تحقیقات بهداشتی  دانشگاه علوم پزشکی تهران  تهران  ایران.**
2- **مرکز تحقیقات عوامل عفونی و بخش زیست شناسی پزشکی  دانشگاه لاوال  کبک  کانادا.**
3- **مرکز آموزش و پژوهش بیماری های پوست و جذام  دانشگاه علوم پزشکی تهران تهران  ایران.**

**خلاصه**

**ضرورت اجرا: بر اساس مطالعات سالهای اخیر تعداد بیماران ایرانی مبتلا به لیشمانیوز جلدی که به مگلومین آنتیمونیت (گلوکانتیم)  اولین خط درمان دارویی در ایران پاسخ مطلوبی نداده اند رو به افزایش است. این مطالعه جهت تعیین ارتباط بین پاسخهای بالینی (بهبود یا عدم بهبود) با میزان حساسیت انگل های *لیشمانیا تروپیکا* به گلوکانتیم طراحی شد.**

**روش ها و یافته ها: در ابتدا میزان حساسیت 185 ایزوله انگل با مدل درون سلولی ماکروفاژ صفاقی موش بررسی گشت. ارتباط کاملا معنی داری بین پاسخ بالینی و مقادیر EC _50_ بدست آمده مشاهده گردید.**

**EC _50_ انگل های جدا شده از زخم بیماران بهبود نیافته  حداقل چهار برابر EC _50_ انگل هایی بود که از زخم بیماران بهبود یافته جدا شده بودند. گزیده ای از این ایزوله ها توسط ژل های پالس فیلد و نیز سکانس ژن پتریدین ردوکتاز1 (PTR1 ) در سطح مولکولی طبقه بندی شدند. این روش ها نشان دادند که 28 ایزوله از مجموع 31 ایزوله انتخاب شده  *لیشمانیا تروپیکا* و بقیه *لیشمانیا ماژور* بودند. ایزوله های *لیشمانیا ماژور* یک گروه متمایز پالس فیلد را تشکیل می دادند و ایزوله های *لیشمانیا تروپیکا* در سه گروه مرتبط پالس فیلد دسته بندی گردیدند. از هر دسته کاریوتایپ پالس فیلد  انگل های حساس و مقاوم انتخاب شدند و برای بررسی بیشتر میزان حساسیت ایزوله های میدانی با مدل رده سلولی مونوسیت THP1  نشانگر لوسیفراز کرم شب تاب وارد انگل ها گردید. این یافته ها به طور خطا ناپذیری تایید نمودند که بیماران با زخم های بهبود نیافته  آلوده به انگل های *لیشمانیا تروپیکا*ی مقاوم به گلوکانتیم بودند. مطالعات بعدی بر روی ایزوله های مقاوم نشان داد که مقاومت پایدار بوده و توسط یک ممانعت کننده بیوسنتز گلوتاتیون به نام بوتیونین سولفوکسیمین میتوان مقاومت را معکوس نمود.**

**بحث : این اولین گزارش انگل هایی با مقاومت ثابت شده است که در شکست درمان لیشمانیوز جلدی سهیم می باشند. همچنین بیانگر وجود ایزوله های میدانی ای در ایران است که به طور اولیه نسبت به گلوکانتیم مقاوم اند.**

**کلمات کلیدی:  *لیشمانیا*  مقاومت داروئی  ترکیبات پنج ظرفیتی آنتی موآن .**
